# Overview of common practices in calf raising facilities

**DOI:** 10.1093/tas/txab234

**Published:** 2022-01-07

**Authors:** Vinicius S Machado, Michael A Ballou

**Affiliations:** Department of Veterinary Sciences, Texas Tech University, Lubbock, TX, USA

**Keywords:** beef-on-dairy, BRD, calf ranch, calves, pre-weaning period

## Abstract

In this literature review, we overview some of the common management practices associated with calf rearing in specialized operations of the United States. Given the growing importance of dairy-beef calves entering the beef production of the United States, we overview aspects related to housing, nutrition, and health events during the pre- and post-weaning period. Based on data on dairy animals, we hypothesize how early life experiences could impact the feedlot performances of dairy-beef animals. Most of the large calf raising operations, where the majority of dairy-beef animals are raised, are located in the Central Great Plains and West regions of the United States. Approximately 80% of calves are individually housed, but the type of housing (e.g., outside hutch, inside a barn) varies based on location of calf-raising facilities. Milk-replacer is fed in more than 80% of operations, while milk (saleable or nonsaleable) is fed in approximately 30% of calf raising facilities (some operations fed more than one type of liquid diet). In addition to liquid feed, water and calf starter are offered ad libitum to calves. Adequate starter intake at weaning is crucial for feed transition from pre- to post-weaning period, which occurs at approximately 2 months of age. Then, calves are mainly housed in group pens and transition from calf-starter to total mixed ration (TMR). Health challenges such as scours and bovine respiratory disease (BRD) can hinder the performance of calves and are major causes of morbidity and mortality in calf ranches. Transportation at a very young age and comingling with animals from other dairies can increase the risk of diseases. Current research efforts are focusing on determining individual factors such as body weight (BW) at arrival or biomarkers of inflammation and stress that can be predictive of disease morbidity, mortality, and performance of calves. Future research should focus on how to utilize this information to optimize management and to develop targeted preventative strategies to reduce incidence of diseases and mortality and improve performance during the pre-weaned period. Also, more research is needed to understand how colostrum management, housing, and nutrition can impact the adult performance of dairy-beef animals.

## INTRODUCTION

Cattle with dairy genetics significantly contributes to global beef production, with dairy herds contributing directly or indirectly with more than half of beef production in European countries (Berry, 2021). In the United States, approximately one-fifth of beef production comes from animals of dairy herd origin (DelCurto et al., 2017). Official reports indicated that almost 10% of beef produced in the United States in 2020 originated from culled dairy cows (USDA, 2020). Additionally, calves born in dairy farms greatly contributes to either veal production or to animals that will be raised exclusively for beef purposes. Generally, the term “dairy-beef” is associated with animals from a dairy origin which end up in the beef production system (either cull cows or calves from dairy herds). For this review, the term dairy-beef will be used to describe animals that are being raised for beef purposes only (bull calves from dairy herds and beef-on-dairy calves, see later); hence, it will not include cull dairy cows.

The practice of raising calves of dairy origin for beef purposes is not new. Many reports from several decades ago provide evidence that the scientific community was already questioning what role that dairy genetics would play in carcass quality and growth performance of beef animals (Judge et al., 1965; Pahnish et al., 1969). However, the importance of dairy originated animals to the beef industry have increased over the past few years. On the beef side, severe droughts significantly impacted cow-calf operations, which led to a decrease in the supply of beef calves. On the dairy side, recent advances in genetics and management increased the fertility and longevity of dairy cows, leading to less demand for raising replacement heifers (De Vries, 2017; Overton and Dhuyvetter, 2020). Additionally, with the advent of sexed semen, producers can inseminate their higher genetic potential animals to provide their replacement heifers, allowing lower genetic animals to be bred to beef semen (De Vries et al., 2008; Overton and Dhuyvetter, 2020). Combined public pressure and decreased veal consumption are encouraging dairy farmers to increase the value of their dairy calves that they do not intend to raise for dairy purposes (Berry, 2021). This has increased the supply of dairy × beef cross calves, also known as beef-on-dairy calves, to enter the beef production chain. Hence, many feedlots are heavily relying on beef-on-dairy animals to meet the increasing consumer demand for beef (Greenwood, 2021). One of the biggest challenges related to beef-on-dairy production systems is the decreased primal cut yields when compared to pure beef animals (Berry, 2021). Although it is recognized that genetic merit plays a major role in the finishing performance of feedlot cattle, it has been suggested that early life experiences may also play a crucial role on how beef-on-dairy animals and pure-bred dairy steers will perform in the feedlot (Twomey et al., 2020).

Therefore, the goal of this review is to overview common practices in calf raising facilities in the United States, and how these practices may impact calves’ health and performance. It is important to highlight that although the practice of raising calves from dairy herds for beef purposes has been done for decades, management practices in many calf ranches are still focused on the goal of raising replacement heifers. Herein, we will highlight how some of these practices optimizing health and growth of calves are associated with productivity of lactating animals, and we will hypothesize how these practices could impact performance of dairy-beef cattle in the feedlot.

## CHARACTERISTICS OF CALF RAISING FACILITIES

### Geographic Location, Size, Animal Sources, and Record Keeping

In the United States, 1 in every 10 dairy replacement heifers (and most bull calves) are raised in an off-site calf raising facility, with at least half of farms with 500 or more lactating animals raised some of their calves in these specialized operations, also known as “calf ranches” (USDA-NAHMS, 2007). These facilities can be geographically distant from the source dairies, which may require long-distance transportation of calves at a very young age, often within the first week of life. The most recent and comprehensive survey describing the characteristics of calf raising facilities in the United States was published about a decade ago (USDA-NAHMS, 2011), and it included 288 operations. Twenty-five percent, 48.6%, and 29.4% of calf raising facilities raised less than 100 (small), between 100 and 999 (medium), and more than 999 calves (large), respectively. Although most operations (80.3%) were in the East region of the country (Indiana, Iowa, Kentucky, Michigan, Minnesota, Missouri, New York, Ohio, Pennsylvania, Vermont, Virginia, and Wisconsin), most of the animals (68.9%) were raised in Western states (Arizona, California, Colorado, Idaho, Kansas, New Mexico, North Dakota, Texas, and Washington), because most large herds were in the West part of the United States (USDA-NAHMS, 2011). With the continuing consolidation of the dairy and beef industry, it is likely that many small and medium operations went out of business since this survey was conducted, which may have increased the proportion of calves raised in large herds. In fact, it is not uncommon to see operations feeding over 20,000 pre-weaned calves in the Central Great Plains and West regions (Moore et al., 2002; McConnel et al., 2019; Celestino et al., 2020).

Most dairies that send their heifer calves to off-site calf raising facilities retain ownership of their animals (USDA-NAHMS, 2014), but over 30% of calf-raising facilities purchase calves from dairies or auction market/sale barns, that do not end up being re-sold to the dairy of origin (USDA-NAHMS, 2011). Additionally, approximately 25% of calf raising operations receive animals from more than five different sources. If only considering large operations, almost half of calf raising facilities receives calves from more than five different sources. Sources include dairy farms, auction markets/sales barns, other calf raising facilities, and private sales not associated with a dairy farm (USDA-NAHMS, 2011). The majority of heifer raising facilities do not receive or purchase pre-weaned calves. About 65% of operations receive weaned animals, whereas only 35% of facilities raise pre-weaned calves. For operations that receive pre-weaned calves, the average age of arrival is approximately 3 days (USDA-NAHMS, 2011; Walker et al., 2012).

One of the most remarkable features of calf raising operations, especially compared to the average cow-calf operation (USDA APHIS, 2011), is animal identification and record keeping. Virtually, all animals in calf raising facilities have at least one type of identification, with more than 60% of herds requiring at least two forms of identification (83.3% if only considering large herds; USDA-NAHMS, 2011). Roughly one-third of calf raising operations receive animals with radio-frequency identification (RFID) ear tags, whereas another 10% of operations insert RFIDs in animals they receive (52.3% and 26.6%, respectively, if considering only large operations; USDA-NAHMS, 2011). Moreover, approximately 75% of operations maintained at least one type of herd ID, allowing them to track the dairy of origin of each particular animal (USDA-NAHMS, 2011). This level of detail in identification of animals allows for in-depth traceability and record keeping, which is of extreme value in supply chains that require or would like traceability. Some of the information recorded in calf ranches include health events, treatments, and growth of calves. Although record keeping was reported to be not routinely practiced among small herds, almost 90% of large operations utilized database software to maintain records. About 75% of calf raising facilities that did not own all their animals share individual animal records with the dairy of origin. Additionally, more than 80% of operations share individual animal information about health, performance, and breeding history to their clients (dairy of origin) or buyers (USDA-NAHMS, 2011). Such information of individual animals can be crucial for the optimization of colostrum management, nutrition, and vaccination protocols.

### Morbidity and Mortality

Considering the economic losses related to morbidity and mortality, and the short- and long-term impact of diseases on performance of calves, it is not surprising that calf health and mortality were listed as the biggest challenges faced by calf raising operations, ranking above feed costs and labor-related issues (USDA-NAHMS, 2011). The most prevalent diseases affecting calves raised in calf ranches are diarrhea (also known as scours) and bovine respiratory disease (BRD), with most of the morbidity observed during the pre-weaning period (USDA-NAHMS, 2011; Walker et al., 2012). The percentage of calves affected with gastrointestinal issues and BRD during the pre-weaning period was 25.3% and 18.1%, respectively. Meanwhile, diarrhea becomes very uncommon after weaning (less than 1% of calves are affected), but respiratory issues are still relevant, with more than 10% of calves being affected by BRD in the post-weaning period (USDA APHIS, 2011). At weaning, calves move from individual hutches/pens into group pens (see later). With the added stress of weaning and comingling, it is common to observe an outbreak of BRD days after weaning, due calf-to-calf transmission of respiratory pathogens and stress-related immunosuppression (Gorden and Plummer, 2010). During the pre-weaning period, failure of passive transfer (FPT) of maternal immunoglobulins from maternal colostrum is likely one of the largest contributors to susceptibility of calves to pathogens (see later), with diarrhea and BRD being linked to poor colostrum management (Godden et al., 2019).

Preventative strategies such as vaccination, metaphylaxis, and other prophylactic strategies, including the use of nutraceuticals are routinely adopted by calf raising operations (Ballou et al., 2019). For instance, in calf ranches located in the West, almost 90% of their calves are either vaccinated prior to arrival or while in the facility (USDA-NAHMS, 2011). Vaccination protocols include immunization against respiratory and gastrointestinal pathogens, both viruses and bacteria. Moreover, metaphylactic use of antimicrobial drugs to prevent diarrhea or BRD occurred in approximately half of calf raising facilities, targeting periods of high susceptibility to diseases (Walker et al., 2012). This practice is known to improve the respiratory health of high-risk pre-weaned calves, especially when they are comingling in group pens (Teixeira et al., 2017a; Bringhenti et al., 2021). Metaphylaxis is also known to mitigate BRD in calves after transportation stress (Duff and Galyean, 2007). However, findings from a recent study suggested that metaphylaxis may not be needed in situations where calves are transported to a calf raising facility and placed in individual hutches and/or when herd morbidity and mortality are low (Celestino et al., 2020).

Similarly to morbidity, mortality is greater among pre-weaned calves in comparison to weaned counterparts, with mortality being 4.2% and 1.6% during the pre- and post-weaning period (USDA-NAHMS, 2011). Most of the mortality, in both periods, is due to BRD, being followed by digestive issues (USDA-NAHMS, 2011), although others reported that at least in the pre-weaning period, scours was the major contributor to mortality among calves raised in off-site facilities (Walker et al., 2012).

As mentioned earlier, most of the management practices and benchmarks are related to the goal of successfully raising replacement heifers. The goal is to raise heifers that will be bred at 13 to 14 months of age [50% to 55% of their mature body weight (BW)] and join the milking herd at approximately 24 months of age (Gabler et al., 2000; Ettema and Santos, 2004). However, many calf ranches raise calves to enter the beef production chain as feeder calves, often mixed with replacement heifers (Walker et al., 2012), but sometimes solely raising bull calves (Moore et al., 2002). Generally, the goal of a calf ranch, regardless of raising replacements heifers or calves that will enter the beef production system, is to provide the adequate environment for calves to promote comfort, health, and growth (McFarland, 2012). However, calf raising facilities are not standardized, and there are many different variations in housing, nutrition, and other management strategies that can play a role on health and performance of calves. These variations are also related to the stage of life of the calf. Therefore, we will review how these different management strategies during the pre- and post-weaning periods can impact the health and performance of calves.

## THE PRE-WEANING PERIOD

While beef calves originated in cow-calf operations suckle in their dams until approximately 8 months of age, calves from dairy herds are weaned around 2 months of life, switching to a complete solid diet after that. The pre-weaning period is the most challenging stage of life of calves born in dairies. They undergo many stressors such as early life transportation, bucket or bottle feeding training, disbud, and castration, with subsequent weaning and commingling with other calves (Hulbert and Moisá, 2016). Hence, it is not surprising that morbidity and mortality are greater during the pre-weaning period than after weaning (USDA-NAHMS, 2011; Walker et al., 2012). Additionally, growth and health during the pre-weaning period have long lasting effects on the adult life performance of dairy animals. For instance, it was reported that for every 1 kg of pre-weaning average daily gain (ADG), heifers produce, on average, over 800 kg more milk in their first and second lactation (Soberon et al., 2012). Moreover, BRD during the pre-weaning period was associated with lower BW gain until 400 days of age (Hurst et al., 2021), reproductive losses, and increased culling prior to first lactation (Teixeira et al., 2017b). Sharon et al. (2019) reported that calves that were previously fed greater quantities of milk solids during the pre-weaning period had improved responses to a BRD challenge 1 month after weaning. Growth and health of calves originated from dairy herds during the pre-weaning period is impacted by many factors, including nutrition, management, and environment. Hence, management practices related to colostrum management, housing, and nutrition during the pre-weaning period may also have long-term impact on the performance of dairy-beef animals.

### Colostrum Management

Most calves born in dairies are separated from their dams immediately after birth, and receive colostrum via a nipple bottle or esophageal tube (Urie et al., 2018). The general recommendation is to feed 10% of BW of good quality colostrum within the first 4 h of life (Godden, 2008). This strategy is known to reduce FTP, historically described as calves that have less than 10 g/L of serum IgG [or 5.2 g/dL of total serum protein (TSP)] after colostrum feeding (McGuirk and Collins, 2004). Generally, colostrum is administered to calves at the dairy of origin. Indeed, all calf raising facilities that participated in the 2011 USDA survey reported that colostrum was administered to calves at the dairy of origin, prior to shipment to calf ranches (USDA-NAHMS, 2011). In addition, approximately 20% of calf ranches reported that colostrum was also administered at their facilities. However, the value of feeding colostrum at the calf ranch if it was not fed within the first 12 h of life is questionable, as many of those immunoglobulins will not be absorbed by the calf at that time. The effect of feeding colostrum after day 1 of life may be more related to development of gastrointestinal integrity or immediate local protection against enteric pathogens by the immunoglobulins in the gastrointestinal tract. Calves that are FPT are more susceptible to diseases and are more likely to die during the pre-weaning period than calves with adequate passive transfer (Godden, 2008). A common benchmark within the dairy industry for proper colostrum management is to have less than 10% of calves experiencing FPT (McGuirk and Collins, 2004). However, it has been proposed that transfer of passive immunity should be categorized as: excellent (≥25.0 g/L of IgG or TSP ≥ 6.2 g/dL), good (18.0 to 24.9 g/L of IgG or TSP = 5.8 to 6.1 g/dL), fair (10.0 to 17.9 g/L of IgG or TSP = 5.1 to 5.7 g/dL), and poor (≤10.0 g/L of IgG or TSP ≤ 5.1 g/dL). To set higher standards for colostrum management within the dairy industry, it was proposed that more than 40% and less than 10% of calves should be in the excellent and poor categories, respectively (Godden et al., 2019).

Therefore, monitoring passive transfer is a very important tool to assess colostrum management quality and understand the potential risk of morbidity and mortality of pre-weaned calves. It seems that calf raising facilities truly understand the value of this practice, as more than 40% of all operations routinely assess passive transfer (USDA-NAHMS, 2011; Walker et al., 2012). If we only consider large operations (raising more than 1000 calves), more than 70% of them routinely assess passive transfer in their calves (USDA-NAHMS, 2011). It is likely that the proportion of operations that routinely evaluate passive transfer has increased over the last decade. For instance, the percentage of dairy farms that adopted that practice increased from 2.1% to 6.2% (14.5% to 38.3%, if considering herds that milk more than 500 cows) from 2007 to 2014 (USDA-NAHMS, 2007; USDA-NAHMS, 2014). Also, it is important to highlight that calf raising facilities routinely assess passive transfer in much greater proportion than dairies that raise their own calves. Historically, bull calves born on dairy farms were deprived of good quality colostrum, as that resource was destined to the higher value female, replacement calves, which represented the future producers of the herd and justified such investment. Hence, off-site calf raising operations would often receive many dairy-beef calves with poor passive transfer status, which affected their business. For instance, herds with a high proportion of FPT are expected to have increased economic costs related to morbidity (labor and treatments cost) and mortality of calves during the pre-weaning period (Raboisson et al., 2016). Hence, most calf raising facilities may either refuse to receive calves based on their passive immunity status or accept them with conditions (USDA-NAHMS, 2007). In our own experience, we have observed that some calf raising operations may pay premiums to dairy farmers based on their calves’ passive immunity status, but it is unclear if that is a widespread practice.

Colostrum pooling is a common practice in the dairy industry (Urie et al., 2018), which allows farmers to build a colostrum bank of adequate quality and availability to feed calves soon after they are born. Therefore, it is not always that calves receive the colostrum of their own dams. This can be an important practice to ensure that all calves receive a good quality colostrum, regardless of how good the colostrum of their dam was. There are several cow-related factors that can impact colostrum quality, such as breed, and perhaps parity (Weaver et al., 2000; Godden et al., 2019; Mcgee and Earley, 2019). Usually it is expected that colostrum quality increases with the age of the cow, although this might not always be the case (Weaver et al., 2000; Godden et al., 2019). Recently, it was reported that calves that received colostrum from their first parity dams had increased passive transfer than calves that received colostrum from their multiparous dams (Shivley et al., 2018). This finding suggests that calves from primiparous dams have improved capacity of IgG absorption even though the concentration of IgG in the colostrum may be less than multiparous cows. Other calf-related factors such as BW at birth have also been associated with colostrum IgG absorption (Teixeira et al., 2013). Because there are differences in colostrum quality within dairy breeds (Godden et al., 2019) and between dairy and beef breeds (Mcgee and Earley, 2019), it is possible that there are breed differences in IgG absorption by the calves. With the widespread use of beef semen in dairy cows, and more beef-on-dairy animals being born, more research is needed in this area to establish if passive transfer efficiency is different between dairy × dairy and beef-on-dairy calves. This could indicate whether the standards set to passive immunity for dairy calves are also applicable to beef-on-dairy animals.

### Housing

Most calves raised in off-site rearing operations are housed individually during the pre-weaning period (USDA-NAHMS, 2011; Walker et al., 2012). However, there are major regional differences in the type of individual housing utilized by calf ranches. For instance, all calves in the West are raised in individual pens, with more than 90% of operations having their individual hutches/pens outdoors. In the East region of the United States, approximately 80% of facilities raise their calves individually. Additionally, in the East, about 30% of the facilities have their individual hutches/pens outside, while almost 45% of facilities have their individual pens inside a barn (USDA-NAHMS, 2011).

Moreover, there are many different designs of individual housing of dairy calves utilized in calf raising facilities (Moore et al., 2012). For example, hutches can be built either from plastic or wood. Regardless of the material, hutches should provide proper ventilation. Usually, plastic hatches have windows and a ridge vent, while many wooden hutches are elevated, which also helps to maintain a clean and dry environment. In nonelevated hutches (wood or plastic), bedding, especially straw or shavings, are utilized to keep calves warm, clean, and dry during colder months (Lago et al., 2006). Many facilities add a small pen adjacent to the hutch or a tether, which provides more space allowance for the calves. Allowing more space per calf was associated with decreased bacterial concentrations in pen/hutch area, leading to improved health outcomes (Lago et al., 2006). Although there are no published data on how these different designs of individual housing are adopted by calf raising facilities around the country, based on our experience, wooden hutches are more prevalent in the Western region.

Traditionally, producers raise calves individually as a strategy to prioritize biosecurity. In this type of housing, the contact between calves is minimized, which leads to decreased cross-contamination and horizontal transmission of pathogens (Barrington et al., 2002). Additionally, monitoring for feed intake (liquid feed or calf starter) and signs of diseases (for instance fecal consistency) can be difficult when calves are housed in group pens. However, recent studies have shown that there are some benefits to house pre-weaned calves in groups or pairs. For instance, calves housed in pairs or in group pens have the opportunity for early socialization, which can reduce stress during weaning and subsequence comingling during the post-weaning period (Cobb et al., 2014a; Cobb et al., 2014b; Costa et al., 2016). Additionally, calves reared in pairs soon after birth have increased solid feed intake compared to calves raised individually or calves paired approximately at 43 days of age (Costa et al., 2015). Moreover, calves reared in groups with three calves per pen consumed more starter after weaning and had increased ADG at the end of the pre-weaning period, with this benefit lasting throughout the immediate post-weaning (Cobb et al., 2014b). This indicates that early socialization is important for the feeding behavior development of calves, which can result in improved weight gains during the pre-weaning period (Costa et al., 2015; Knauer et al., 2021). Timing of socialization seems to be very important for benefits in solid feed intake. When socialization did not occur soon after birth, it did not result in benefits for feed intake and weight gain (Bučková et al., 2021). Also, stress reduction during weaning was improved for calves grouped at 5 days of age in comparison to calves socialized at 28 days of life (Bolt et al., 2017). Raising calves in group pens can reduce labor, as the implementation of automatic feeders becomes a possibility in this system. More technologically advanced automatic feeders can monitor individual feed intakes, which can aid in the detection of disease and behavior monitoring (Bowen et al., 2021); however, the capital investment for automatic feeding systems at a large scale may be unfeasible due to logistics and cost.

One of the major concerns of pair or group housing during the pre-weaning period is the transmission of pathogens and possible increase in morbidity and mortality. Cobb et al. (2014a) reported that calves raised in either pairs or groups of three calves in a poor indoor environment had a tendency for an increased incidence of BRD during the first 90 days of life when compared to calves raised individually in the same barn. However, other data demonstrated that, at least when calves are pair-housed, the frequency of respiratory problems and gastrointestinal disorders were not different when compared to calves housed individually (Bučková et al., 2021; Knauer et al., 2021). However, some studies focused in respiratory disease in pre-weaned calves grouped in pens of 20 animals have reported high morbidity (Teixeira et al., 2017a; Bringhenti et al., 2021). Although there is no evidence supporting that respiratory issues experienced by those calves were due to group housing, this is still a great concern of calf raisers.

Collectively, we can conclude that there is a growing body of evidence showing that raising pre-weaned calves in pairs or groups can improve their social environment, leading to increased solid feed intake and weight gain. However, we should expect that the adoption of this housing system will be slow and gradual in calf raising operations. For instance, adoption of group housing during the pre-weaning period in dairy farms that raised their own replacement heifers has not differed significantly in the last decade; still around only 20% of dairies raising their calves in group pens (Urie et al., 2018). For calf raising facilities, a transition from individual housing to group housing can be even more challenging. In some cases, especially when the operation utilizes wooden hutches, this transition would require an enormous capital investment, that could be unfeasible to the operation. For operations that utilizes plastic hutches, it has been offered some options on how to transition to a pair housing system. It was proposed that pair housing using individual hutches may represent an alternative to individual housing (Wormsbecher et al., 2017). In that study, calves were either individually housed in a 2.4-m^2^ plastic hutch with access to an outside area of 6.9 m^2^ or were housed in pair, having access to two hutches attached to 13.7 m^2^ of outdoor space; therefore, space allowance per calf was not different but two calves were able to socialize together. Wormsbecher et al. (2017) demonstrated that this pair housing system allowed calves to socialize and interact, even spending time inside of the same hutch. However, no differences in growth were observed, and starter intake was not reported. It is important to highlight that the study was designed to avoid competition of resources, and 16 L/d of milk was offered to the calves, which was essentially ad libitum, as daily milk intake never reached 16 L/d. As we will review later in this article, this is a much greater milk allowance than what most calf ranches offer. Based on other studies (Cobb et al., 2014b; Costa et al., 2015; Knauer et al., 2021), it would be expected that starter intake would be increased for pair-housed calves, leading to increased growth in more restricted liquid feed programs. Indeed, in another study where a similar pair housing strategy (doubling the hutch and outdoor space) was tested. Although differences in growth were not observed, calves housed in pairs had increased starter intake during the pre-weaning period and had a better transition to the post-weaning diet (Whalin et al., 2018). Although Whalin et al. (2018) reported that cross sucking only occurred five times during the pre-weaning period in calves housed in pairs, this abnormal behavior could result in navel and ear abscesses (Mahmoud et al., 2016). Feeding milk through slow-flow teats can aid in avoiding cross suckling in calves housed in pairs (Salter et al., 2021). Then, information on how to transition from individual plastic hutches to an adapted pair housing system is available. However, it is important that calf raising facilities understand that these studies were done with a very limited number of animals. To perform such transition in large scale, with some herds having many thousands of individual hutches, will be difficult and will require further training of the operations’ staff. In addition to improved perceived welfare of calves and improved performance, societal pressure may also play a role in the motivation to transition calves from individual to pair-housing systems (Ritter et al., 2021).

As mentioned earlier, Twomey et al. (2020) suggested that the differences in feedlot performance between conventional beef and beef-on-dairy animals is likely due to different early life experiences. It was alluded that group housing more closely resemble the natural and semi-natural social environment observed in cow-calf operations, where calves are raised with their dams and other calves (Cantor et al., 2019). However, more research is needed to investigate the long-term impacts on performance that group or pair housing could have in dairy-beef calves.

### Nutrition

Proper nutrition is a major pilar supporting health and growth of pre-weaned calves, having long-lasting impacts in the performance of calves raised for dairy purposes (Soberon and Van Amburgh, 2013; Gelsinger et al., 2016; Sharon et al., 2019). In cow-calf operations, calves can suckle their dams throughout their pre-weaning period, which usually lasts up to 8 months. Hence, milk and pasture provide the needed nutrition that supports growth and ruminal maturation in those circumstances. In calf raising facilities, calves have a much shorter pre-weaning period. Thus, the goal of a nutritional plan in a calf ranch is to provide nutrients to promote adequate growth and support immunity, while stimulating ruminal development for calves to be weaned around 60 days of life. Here, we will discuss the different nutritional strategies related to liquid and solid feed provided by calf raising operations during the pre-weaning period.

In many cases, calf raising facilities may feed more than just one type of liquid feed. For instance, some operations, depending on availability, may feed their calves a mix of milk replacer (or milk components blended together on farm to make their own milk replacer; collectively we will refer to both these as milk replacers) and whole or nonsaleable milk. Almost 90% of operations utilized at least some milk replacer to feed their calves, with most of them feeding medicated milk replacers for at least part of the pre-weaning period (USDA-NAHMS, 2011). Additionally, 67.9% of operations fed only milk replacers (USDA-NAHMS, 2011). It is possible that after the new Veterinary Feed Directive became effective in 2017, the use of medicated milk replacers may have decreased. However, despite over the counter purchases of medicated milk replacers are no longer allowed, more than 75% of calf raising facilities maintain a strong presence of veterinarians in their operations (USDA-NAHMS, 2011). Hence, these operations are able to purchase medicated milk replacers if prescribed by their veterinarians. Among the operations that fed milk replacers, more than 80% fed protein and fat contents between 20% and 24% (USDA-NAHMS, 2011). The major sources of proteins in milk replacers are still predominately from milk sources, including whey protein concentrate or nonfat dried milk. Additionally, the use of animal plasma from either bovine and/(or) porcine sources is common in calf ranches, at least during the first few weeks of life, where 20% to 30% of the protein may come from plasma (BAMN, 2008). The partial replacement of milk protein sources with vegetable proteins, including soy protein isolates and hydrolyzed wheat gluten, are available; however, the specific use by calf ranches is not known. The fat is commonly either protein encapsulated, sprayed on to the milk powder during the manufacturing a dried commercial milk replacer, or added to the liquid diet at the calf ranch as a liquid fat. The sources of fat vary, but are commonly a blend of animal fats, e.g., lard and tallow, but can also include some desired vegetable fat, e.g., coconut. The fatty acid composition of coconut fat contains a high proportion of medium chain fatty acids, which may have some antimicrobial activity, but are also highly digestible by the calf. Lastly, carbohydrates sources, e.g., 99% lactose or sweet whey protein, are commonly added to balance the liquid diet of the calves. Most of the liquid diet programs also include a vitamin, both fat and water soluble, and trace mineral supplement that is either added in the commercial milk replacer or on-farm when mixing the liquid diet. About one-third of facilities included whole milk in their liquid diet. Unsaleable milk was pasteurized in most operations prior to feeding (Walker et al., 2012). Two thirds of calf ranches that included whole milk in their nutritional strategy sourced milk from multiple dairies, with a fraction of operations from the West also sourcing rejected milk from processing plants or expired milk products from grocery markets, also known as “squeeze milk” (USDA-NAHMS, 2011).

All small and medium operations fed liquid diets twice daily, while 14.3% of large facilities opted to feed three times per day. Additionally, 74% of operations fed between 1.9 and 2.8 L of liquid feed per feeding, with the remaining of herds feeding more than 2.8 L per feeding. Hence, 70.4%, 21.1%, and 8.5% of operations feed 3.8 to 4.7 L, 5.7 to 6.6 L, and more than 6.6 L of milk replacer and/or milk per day. Liquid feed is mainly fed in either buckets or nipple bottles, with a major discrepancy by region. Operations from the West mainly utilize bottles, while bucket is the preferred equipment to feed calves in the East. Additionally, routine disinfection of feeding equipment is adopted by all operations located in Western states, being less frequent in the East (USDA-NAHMS, 2011).

In addition to milk or milk replacer, the inclusion of solid feed into nutritional programs of pre-weaned calves is crucial to meet nutritional requirements for proper growth and ruminal development (Niwińska et al., 2017). The formation of volatile fatty acids, especially butyrate, plays an important role stimulating ruminal maturation, such as development of ruminal papillae, increase in the number of ruminal bacteria and protozoa, and increase of motility (Khan et al., 2016). Therefore, the recommendation is to provide pre-weaned calves unrestricted access to water and a calf starter from a very young age, preferably since the first day of life (Jones and Heinrichs, 2003; Amaral-Phillips et al., 2006). The average days of age when calves were offered water and calf starter in calf raising facilities was 6.6 and 6.3 days, respectively; and for large herds, it was 4.1 and 3.5 (USDA-NAHMS, 2011). Modern calf starter diets include between 18% and 22% of crude protein, while NDF, nonfiber carbohydrate, and starch levels can range between 15% to 20%, 50% to 55%, and 35% to 40%, respectively. Calf starter is usually offered as a complete pellet or in texturized form. Forage is not commonly added to calf starter, and when included, it commonly represents less than 5% of the dry matter and is finely chopped. In fact, hay or other roughages are only include in the diet of calves later in life. In large operations, hay or other roughages were only offered to calves after weaning, at 70 days of life (USDA-NAHMS, 2011).

One of the benchmarks used to evaluate the success of calf raising strategies is assess calf starter intake at weaning. Commonly, it is expected that calves go into the post-weaning period eating at least 2 lbs (0.91 kg) of calf starter per day (Amaral-Phillips et al., 2006). Many factors can influence starter intake, such as palatability and water intake (Kertz et al., 1984). As previously mentioned, housing and social interaction at a very young age can also influence the amount of solid feed calves eat during the pre-weaning period (Costa et al., 2015; Knauer et al., 2021). However, perhaps the major driver of starter intake during the pre-weaning period is milk or milk replacer intake/allowance. When calves have unrestricted access to liquid feed, they can drink up to 15 L of milk replacer per day (Curtis et al., 2018). Such a strategy can lead to improved ADG during the pre-weaning period (Jasper and Weary, 2002; Curtis et al., 2018). Additionally, calves fed higher planes of nutrition in milk replacer had improved innate immunity responses after an oral challenge with *Salmonella enterica*, suggesting that more milk replacer intake can reduce the susceptibility of calves to certain pathogens (Ballou et al., 2015). However, increased intakes of liquid feed can dramatically delay and decrease calf starter intake (Curtis et al., 2018) and reduce the efficiency of digesting solid diets at weaning (Hill et al., 2016). Recent findings suggests that some of these pitfalls of feeding high levels of milk or milk replacer could be prevented with gradual weaning or increasing the length of the pre-weaning period (de Passillé and Rushen, 2016; Lopreiato et al., 2018). There seems to be trade-off depending on which nutritional strategy (accelerated or restricted milk feeding programs) a calf raising facility decides to adopt. Generally, feeding solid feed to calves is less expensive than feeding milk or milk replacer. Additionally, considering that feeding lower levels of milk leads to a smoother dietary transition to the post-weaning period, and that most calf ranchers are not compensated based on calf growth and performance, it is not surprising that most operations feed less than 5 L of milk or milk replacer per day (USDA-NAHMS, 2011). Conversely, the amount of milk fed per day by dairy farms that raise their own calves is greater then what is observed in off-site calf raising facilities (Urie et al., 2018). As discussed earlier, there are some potential long-term economic advantages in feeding more milk to calves to accelerate their growth during the pre-weaning period, which will result in long-term benefits in weight gain and lactational performance (Soberon et al., 2012; Sharon et al., 2019; Hurst et al., 2021). However, the long-term impact of accelerated growth during the pre-weaning period on the adult performance of dairy-beef animals is unknown, and more research in this area is warranted. It is important to highlight that financial incentives will be needed for calf ranchers to adopt strategies that would increase the cost of calf rearing, such as increases in milk allowance and extended pre-weaning period.

Some of the major concerns regarding dairy-beef calves is the high prevalence of liver abscesses observed in packing plants (Amachawadi and Nagaraja, 2016). Historically, greater prevalence of liver abscesses in dairy breeds, especially Holsteins, were reported, with Holsteins steers having on average twice as many liver abscesses than beef steers and heifers (Amachawadi and Nagaraja, 2016). However, industry leaders are recently reporting liver abscesses in up to 60% of dairy × dairy and dairy × beef crosses. There may be a genetic component associated to susceptibility to liver abscesses (Keele et al., 2016), with dairy and beef-on-dairy crosses potentially being naturally more likely to develop this disease. However, liver abscesses development is primarily caused by commensal bacteria leaking from the gastrointestinal tract after ruminal acidosis episodes, reaching the liver through the hepatic portal vein (Nagaraja and Lechtenberg, 2007). Thus, it is not surprising that the majority of liver abscesses develops during the finishing period when the high grain based diets are fed to cattle (Nagaraja and Chengappa, 1998). However, anecdotal observations have suggested that many animals from calf raising facilities are entering the feed yards with liver abscesses. Longitudinal research is needed to understand when dairy-beef calves are developing liver abscesses through the course of the production system. Calf starter and grower diets (see later) are more comparable to finishing diets in terms of fiber and starch contents. Hence, the increased liver abscesses prevalence among animals originated in dairies observed in packing plants may be due to high starch and low fiber diets fed from a very young age until slaughter. Some researchers have suggested that increasing effective fiber in calf starter is needed to avoid ruminal acidosis and secondary disorders such as liver abscesses (Khan et al., 2016). However, the starch and effective fiber contents should be optimized to avoid potential delays in growth and ruminal development during the pre-weaning period, which could be cost prohibitive to calf raising operations. Hence, more research in pre-weaning and post-weaning nutrition are needed to investigate its role in the development of hepatic abscesses.

## THE POST-WEANING PERIOD

In calf raising facilities, calves are generally weaned on average at 7 weeks of age (USDA-NAHMS, 2011; Walker et al., 2012), but the average age at weaning is greater in operations located in the West of the United States in comparison to the ones in the East (8.9 vs 6.7; USDA-NAHMS, 2011). After weaning, dairy-beef calves go through an immediate post-weaning stage (from weaning to about 90 days of age), and then a grower stage (from 90 to 150–180 days of age). Dairy-beef calves are expected to weigh approximately 350 lbs (~160 kg) by 150 days of life, ending their time at the calf raising operation and ready to be shipped to a feed yard. Dairy heifers remain in the calf raising facility after the grower phase, going to the “young heifer” stage until approximately 400 days, when they should have attained approximately 50% to 55% of their mature BW to be bred and eventually return to their dairy of origin or be sold to another lactating herd. Here, we will focus on the immediate post-weaning and grower stages, as these are relevant to dairy-beef animals.

As mentioned earlier, most of the emphasis on calf rearing is placed in the pre-weaning period as morbidity and mortality are greater in that stage in comparison to the post-weaning period, and growth and health events have long-term impacts in the performance of lactating animals (Soberon et al., 2012; Teixeira et al., 2017b). However, in addition to impacting the probability of survival until first lactation, it was suggested that BRD occurring after calves are weaned may impact milk yield in the first lactation (Stanton et al., 2012). Additionally, multiple episodes of BRD, including cases occurring after weaning, decreases the probability of animals to reach their second lactation (Bach, 2011). Social and nutritional changes occurring at weaning are considered stressors that may impact the health and performance of calves (Hulbert and Moisá, 2016).

### Housing

While almost all calves are housed individually during the pre-weaning period, virtually all calves are raised in groups after weaning (USDA-NAHMS, 2011; Walker et al., 2012). To avoid additional stressors related to comingling at the same time of the nutritional stress that constitutes weaning, many producers choose to maintain calves housed individually during the immediate post-weaning period (Moore et al., 2012). This will allow calves to cope with only being fed calf starter while still in a familiar environment, without any disturbances related to novel social interactions. Some argue that such transition from pre- to post-weaning period should be done in small groups, of three or four calves (McFarland, 2012). Such grouping could be done in small pens or in oversized plastic hutches, also known as “super hutches” (Moore et al., 2012). Such grouping strategy is more commonly observed in dairies that raise their own replacement heifers. Hence, in calf raising operations, calves are either maintained in their individual hutches for about a month after they are weaned, and then grouped with other calves during the grower stage, commonly in pens with between 10 and 50 calves.

The type of group housing adopted by calf ranches differs based on region of United States where the calf raising facilities are located. Approximately 75% of operations in the West house their weaned calves in dry-lots, whereas only 5.3% of operations from the East utilize this housing system. Bedded pack/open shed, freestalls, and multiple-animal inside barn/shed were the most prevalent housing type for weaned calves adopted by 21.3%, 20.1%, and 18.9% of calf operations from the East, respectively. Only 6.8% and 14.8% of operations from the West and East raised their weaned calves on pasture, respectively (USDA-NAHMS, 2011). It is important to highlight that for outdoor housing systems, proper shading and protection from weather are provided to animals to mitigate either cold or heat stress, whereas indoor housing types should have good drainage and proper ventilation to promote air quality and decrease the transmission of respiratory pathogens (Moore et al., 2012).

### Nutrition

Regardless of housing type, weaned calves should be raised in an environment that minimizes competition to resources. The targeted ADG for calves during the immediate post-weaning and grower stages is approximately 0.9 to 1 kg/day. Therefore, feed and water should be offered ad libitum. As stated earlier, calves are expected to be eating almost 1 kg/day of starter at the time of weaning, as they will solely rely on solid feed for their nutrition. During the immediate post-weaning, most producers continue to feed calf starter. This strategy is thought to mitigate stress related to weaning practices, especially for calves that switch from individual to group housing at weaning (Amaral-Phillips et al., 2006). Hence, calf raising facilities allow calves to eat calf starter for another month, before switching them to a “grower diet”. It is worth noting that over the past few years the authors have observed many calves are being switched to the grower diet at approximately 40 to 60 days of life in the West. Grower rations are usually offered as a total mixed ration (TMR), that contains significantly more fiber than calf starter, and when offered to calves before weaning results in decreased ADG (Hill et al., 2008) The exact reason(s) why we have seen this switch to a grower diet at an earlier age is not completely understood, but the perception is the calves perform well and the risk for bloat is reduced. More research is needed in this area to understand the potential health benefits to switching a grower diet earlier at the expense of body weight growth. High fiber byproducts such as cottonseed hulls, soy hulls, or beet pulp are used in grower diets, with the overall diet crude protein, NDF, nonfiber carbohydrate, and starch contents ranging between at 16 and 18%, 25 and 30%, 45 and 50%, and 35 and 40%, respectively (Akins, 2016).

As reviewed earlier, there are growing concerns related to the occurrence of liver abscesses while calves are still in calf ranches. Perhaps optimization of immediate post-weaning and grower diets are needed to reduce the incidence of liver abscesses during those stages of calf rearing. However, more research is needed to confirm if the greater prevalence of liver abscesses in dairy-beef animals is originating in calf ranches. If this is confirmed, further studies should be conducted with the goal of optimizing pre- and post-weaning diets to avoid the development of liver abscesses without significantly impacting the growth of calves. There is the possibility that animals that receive high energy diets early in life may be more adapted to the high-grain diets commonly fed when they arrive to feedlots. This should also be the subject of further research.

## UTILIZING INFORMATION COLLECTED AT ARRIVAL AT CALF RAISING FACILITY TO ASSESS MORBIDITY AND MORTALITY RISK AND PERFORMANCE

During the transit to a calf raising facility, calves must cope with stresses related to transportation and comingling at a very young age. This added level of stress during the first days of life can lead to immune dysregulation which could result in increased risk of morbidity and mortality, and poor growth performance (Hulbert and Moisá, 2016). Knowing which calves may be at an increased risk for a negative outcome because of transportation related stressors can assist calf ranchers to make individual management adjustments that could improve the calves’ health and performance. Some of these adjustments could be metaphylaxis or other prophylactic treatment. Additionally, high-risk calves could be put in pens/hutches where they could be more frequently monitored for health disorders. Hence, recent research has been performed to evaluate the association of stress, metabolism, and inflammation biomarkers, and information related to health status collected at arrival with the incidence if diseases, mortality, and growth of calves transported to specialized calf rearing operations. Most of the research performed so far was done in Canadian or European veal facilities (Marcato et al., 2018). However, we believe that the similarities between rearing calves for veal or calves that will enter the beef industry may allow for the application that new knowledge to calf raising facilities.

Although calf cleanliness was historically perceived as an indicator of diarrhea in calves, hide cleanliness assessed at arrival to a veal facility was not associated with the risk of diarrhea in calves (Graham et al., 2018). In another study, the same group evaluated the association of several health-related information assessed at arrival with morbidity in veal calves, demonstrating that low BW and high rectal temperature at arrival were associated with an increased hazard of morbidity (von Konigslow et al., 2020; Goetz et al., 2021). Additionally, morbidity in veal calves was associated with biomarkers of metabolism, stress, and inflammation. For instance, von Konigslow et al. (2020) reported that blood lymphocyte counts lower than 7.0 × 10^9^ cells/L was associated with increased morbidity. Additionally, increased blood concentrations of haptoglobin (Hp) and molybdenum, and decreased concentrations of IgG and creatine kinase assessed at arrival were associated with lower risk of disease over 78 days in a veal facility (Goetz et al., 2021). Moreover, calves that arrive with high cortisol and low albumin blood concentrations at a veal facility are more likely to be diagnosed with lung consolidation (Masmeijer et al., 2021).

Mortality in veal facilities was also associated with several variables assessed at arrival. Season of arrival, low body weight, abnormal navel, dehydration, high rectal temperature, and low blood concentrations of glucose, cholesterol, and IgG were associated with increased risk of mortality (Renaud et al., 2018a; Renaud et al., 2018b; Goetz et al., 2021). Additionally, lymphocyte counts between 4.8 and 5.8 × 10^9^ cells/L and neutrophil counts less than 6.0 × 10^9^ cells/L assessed in blood collected 72 h after arrival was associated with decreased risk of mortality (von Konigslow et al., 2020). Many variables were also associated with growth performance of calves in veal facilities. Low BW at arrival and dehydration were associated with low ADG in veal calves (Renaud et al., 2018c; von Konigslow et al., 2020; Goetz et al., 2021). Additionally, biomarkers measured on blood at arrival were also associated with growth. While lymphocyte counts, creatine kinase, cholesterol, iron, copper, and IgG were positively associated with ADG, haptoglobin and zinc were negatively associated with growth in veal calves (von Konigslow et al., 2020; Goetz et al., 2021).

All the results described above were from studies conducted in veal calves. Recently, our group utilized a similar approach to assess the association of biomarkers assessed at arrival with health, mortality, and growth of pre-weaned calves that were transported to a calf raising facility between 3 and 4 days of life (Celestino et al., 2021). In addition to the circulating concentrations of biomarkers assessed at arrival (Hp, cortisol, and l-lactate), other variables were also considered in our analysis (dam’s parity, dystocia, breed, dam’s days dry gestation length, days of age at arrival, birth weight, and total serum protein measured before transportation). We observed that, among all the variables analyzed, Hp was the only variable associated with morbidity, with calves with circulating Hp > 0.63 μmol/L being less likely to develop BRD then calves with Hp ≤ 0.63 μmol/L. Moreover, birth weight was negatively associated with ADG, while jersey-cross calves gained more weight during the pre-weaning period than pure bred jersey calves. It is important to highlight that this study was performed only with female dairy calves, and more research is needed to understand which variables are associated with health, mortality, and performance of pre-weaned dairy-beef animals. It is likely that the identification of high-risk calves based on information collected at arrival will be incorporated into complex algorithms, as the accuracy of variables when standing alone may be too low to have clinical applications (Celestino et al., 2021). Also, more research is warranted to establish how information collected at arrival could be utilized to optimize the management of high-risk calves in calf raising operations.

## SUMMARY

With the increased use of sexed semen, and the decreased needs for replacement heifers, dairy farmers are substantially increasing the supply of beef-on-dairy animals, which are being incorporated into the beef production supply chain. Before they head to feedlots, these animals are transported from their dairy of origin to calf raising facilities, where they are reared for approximately 5 to 6 months of age. The same practices adopted for the rearing of replacement dairy heifers are in place for raising dairy-beef calves in calf raising operations. With a pre-weaning period that lasts on average approximately 2 months, calves are usually housed individually. During this time, calves are more susceptible to disease and mortality. Additionally, calves are fed sufficient quantities of a liquid diet to promote growth and encourage calf starter intake preparing the rumen for the post-weaning period. After weaning, calves a mostly reared in group pens and transition from calf starter to a TMR-based diet. To ensure optimization of this supply chain of calves produced for beef, more research is needed to understand how early life experiences could affect the feedlot performance of dairy-beef animals. The dairy-beef animal is an important animal to the beef production and offer many advantages over traditional beef calf supply chain including, scale, traceability, genetic improvement, consistency, and quality.
